# Syphilitic hepatitis: a case report and review of the literature

**DOI:** 10.1186/s12876-019-1112-z

**Published:** 2019-11-19

**Authors:** Jiaofeng Huang, Su Lin, Mingfang Wang, Bo Wan, Yueyong Zhu

**Affiliations:** 1 Department of Liver Research Center, the First Affiliated Hospital of Fujian Medical University, No. 20, Chazhong Road, Taijiang District, Fuzhou, 350001 Fujian China; 2 Faculty of Life Sciences and Medicine, King’s College London, London, SE1 1UL UK

**Keywords:** *Syphilis*, Hepatitis, Rashes, Enzymes, Infection

## Abstract

**Background:**

*Syphilis* is a common disease that has been researched and focused on for many years, however, syphilitic hepatitis has not been well-recognized. We report this case of syphilitic hepatitis with intrahepatic cholestasis and liver granulomas to make a deeper impression.

**Case presentation:**

A 47-year-old male was admitted with jaundice and rashes. The laboratory examination showed abnormal liver enzymes with significant increases in ALP and GGT but mild increases in ALT and AST. His HBV surface antigen was weakly positive, with negative HIV antibody, HCV antibody, and undetectable HBV DNA. The rapid plasma reagin test and the Treponema pallidum particle assay tests for *Syphilis* were both positive. Abdominal ultrasonography and magnetic resonance cholangiopancreatography revealed the normal biliary tract, liver, and spleen. The liver pathological examination showed cholangiocyte inflammation and micro-granulomas with coagulation necrosis. After 2 months of benzathine penicillin treatment, his liver enzyme decreased rapidly and remained normal after 1-year of follow-up.

**Conclusions:**

Increased liver enzymes, intrahepatic cholestasis and liver granulomas with well-response to antibiotics may provide clues for the diagnosis of syphilitic hepatitis.

## Background

*Syphilis* is a multi-systemic disease caused by *spirochete Treponema pallidum*. Liver is one of the organs that can be affected [1]. Congenital syphilitic hepatitis is more common and easily recognized for its typical clinical features, while acquired syphilitic hepatitis in adults were rarely reported. Here we present a clinical case of syphilitic hepatitis whose diagnosis has been confirmed by the pathology of liver biopsy.

## Case presentation

**Fig. 1:**
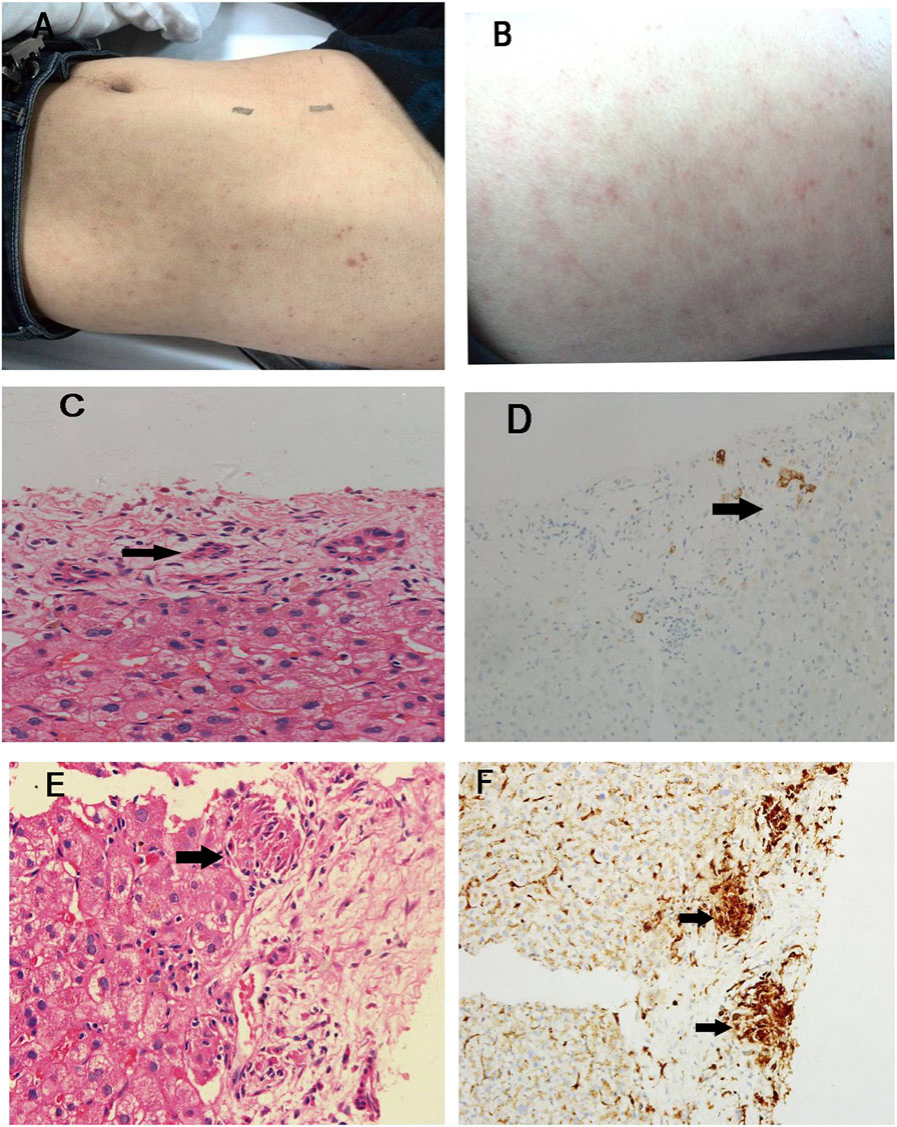
Patient’s body appearance and histological findings. **a** multiple erythematous macules and hyperpigmentation on the back; **b**, multiple erythematous macules and hyperpigmentation over the belly; **c**, HE staining shows bile duct epithelial cell injury, atrophy cholangiocyte, and portal tract inflammation (× 400); **d**, CK7 staining of cholangiocyte reveals atrophy cholangiocyte and bile duct lesion (× 100); **e**, HE staining shows granulomas (× 400); **f**, CD68 staining of macrophagocyte shows granulomas (× 100). Black arrows indicate lesions

A 47-year-old man was hospitalized because of jaundice and rashes. He developed jaundice 2 weeks prior to admission. He had no fever, fatigue, anorexia, abdominal pain, or waist pain. The laboratory examinations in local hospital revealed an alanine aminotransferase (ALT) of 359 U/L, an aspartate aminotransferase (AST) of 161 U/L, an alkaline phosphatase (ALP) of 580 U/L, a gamma-glutamyl transpeptidase (GGT) of 883 U/L and a total bilirubin (TBIL) of 75.1 *u*mol/L. He received a liver protective medication (diammonium glycyrrhizinate), but jaundice did not improve. On the third day of diammonium glycyrrhizinate treatment, multiple non-itchy rashes appeared all over his body, which remained after the withdrawal of diammonium glycyrrhizinate and the application of anti-allergic agents. In addition, his ALP and GGT increased to 853 and 1012 U/L in the fifth day. He was then transferred to our hospital. He denied any chronic diseases, drug administration, alcohol consumption or venereal exposure. Physical examination showed the temperature of 36.0 °C, the blood pressure of 108/75 mmHg, the heart rate of 77 times/min and the breath rate of 18 times/min. His skin and sclera were mildly yellowish. No hepatomegaly, splenomegaly or ascites was found. Non-itchy macular rashes measuring 0.5 to 2 cm were observed all over his body, including some on the soles and palms (Fig. 1a and b). No genital or buccal lesions were found. Biochemical examinations in our hospital revealed a weakly positive in hepatitis B surface antigen (HBsAg). His hepatitis B surface antibody, hepatitis B extracellular antigen (HBeAg) were both negative and his hepatitis B virus DNA was undetectable (< 500 IU/ml). The other laboratory tests including Hepatitis A virus, Hepatitis C virus, Hepatitis E virus, human immunodeficiency virus, Epstein-Barr virus, cytomegalovirus, and other autoantibodies were all negative. Abdominal ultrasonography and magnetic resonance cholangiopancreatography revealed the normal biliary tract, liver, and spleen. He underwent liver biopsy and the results revealed granulomatous hepatitis with stage 2 inflammation and stage 1 fibrosis (Fig. 1c, d, e, and f). Mild hepatic lobule inflammation and plasma cells infiltrating were found in the portal area. In addition, micro-granulomas with coagulation necrosis were noticed in the portal area. Immunohistochemistry examination showed a weakly staining of HBsAg and HBcAg. He received a following test of *syphilis.* The rapid plasma reagin test (RPR) was positive (1,32 titer), and the Treponema pallidum particle assay (TPPA) test was 1:38, which confirmed the diagnosis of *syphilis*. The patient was then given intramuscular benzathine penicillin treatment at a dose of 2.4 million units per week for successive 2 months. No anti-HBV drug was given. The liver enzymes decreased rapidly after penicillin treatment and finally returned to normal level after 2 months of treatment. The follow-up showed that the liver function kept normal and HBV DNA was still undetected after 1-year.

## Discussion and conclusions

Liver injury is often caused by hepatic pathogens infection, while, non-hepatotropic pathogens, such as *Toxoplasma gondii*, *Salmonella*, *Leishmaniasis* and *Tuberculosis* are able to cause the liver injury [2, 3]. *Syphilis* is one of the non-hepatotropic pathogens that cause unidentified hepatitis. Syphilitic hepatitis was first described by Harn in 1943 [4]. In 2004, Mullick [5] proposed the diagnostic criteria of syphilitic hepatitis, which includes:(1) abnormal liver enzyme levels; (2) serological evidence for *syphilis*; (3) exclusion of other causes of liver diseases; (4) liver enzyme levels returning to normal after appropriate antimicrobial therapy. In this case, the patient met all of the above diagnostic criteria.

The clinical manifestations of syphilitic hepatitis in adults tend to be nonspecific and protean [1]. Though rashes and icterus are observed in the majority of patients. The rashes of *syphilis* often present as non-pruritis multiple erythematous and nonconfluent maculopapular lesions, concentrating in trunk, palms, and soles [6, 7]. Other common symptoms include low-grade fever, abdominal pain, phallodynia, sore throat, headache, weight loss, arthralgia or myodynia, splenomegaly, lymphadenopathy, and uveitis [1, 8, 9].

The histological features of syphilitic hepatitis can include bile duct inflammatory infiltration, which may contribute to the elevated ALP and GGT levels in biochemistry tests [1, 7]. Hepatic granulomas are another characteristic of syphilitic hepatitis [3]. Our case presented the typical intrahepatic bile duct inflammation and granuloma, which is consistent with the previously reported cases [9]. In theory, the spirochetes could be identified in liver tissue by immunohistochemical staining or a Warthin–Starry stain [10], however, it was rarely reported in cases published.

Penicillin is the first-line treatment of *syphilis* and the response to antimicrobial therapy is regarded as one of the diagnostic criteria of syphilitic hepatitis [5]. In this case, standard therapy was given where significant improvement was afterward achieved. These further confirmed the diagnosis of syphilitic hepatitis. The Jarisch-Herxheimer reaction (JHR) is a severe immunological phenomenon easily seen in patients during penicillin therapy, and it mainly manifests as short-term symptoms such as fever, headache, myalgias, chills, even a sudden drop of body temperature [11]. Fortunately, JHR did not occur in our patient. According to previous reports, patients who had JHR can also achieve therapeutic effects through dose adjustment or the replacement of antibiotics [12].

In conclusion, there are no specific symptoms for syphilitic hepatitis. Elevated liver enzymes, especially for ALP and GGT, are common in patients. Bile duct inflammation or granuloma formation in hepatic pathology, as well as the response to antibiotic therapy, can also provide some clues for the diagnosis of syphilitic hepatitis.

## Data Availability

The datasets supporting the conclusions of this article are included in the article.

## Abbreviations

ALP: Alkaline phosphatase
ALT: Alanine transaminase
AST: Aspartate aminotransferase
GGT: Gamma-glutamyl transpeptidase
HBV: Hepatitis B virus
HCV: Hepatitis C virus
HIV: Human immunodeficiency virus
JHR: Jarisch-Herxheimer reaction
RPR: Apid plasma reagin test
TPPA: Treponema pallidum particle assay
